# A new Asian leaf litter toad of the genus *Leptobrachella* (Amphibia, Anura, Megophryidae) from northeastern Guizhou, China

**DOI:** 10.3897/zookeys.1295.206395

**Published:** 2026-10-19

**Authors:** Zhaohui Yang, Jing Liu, Fan Zhang, Yanlin Cheng, Qixian Zou, Yunlong Chen, Lang Mu, Junjian Zhou, Shize Li, Heng Zhao

**Affiliations:** 1 Guizhou Mayanghe National Nature Reserve Administration, Yanhe 565300, China Guizhou Mayanghe National Nature Reserve Administration Yanhe China; 2 Guizhou Wildlife Conservation Association, Guiyang 550025, China Guizhou Wildlife Conservation Association Guiyang China; 3 College of Resources and Environment, Moutai Institute, 564500, Renhuai, China College of Resources and Environment, Moutai Institute Renhuai China; 4 College of Life Science, Guizhou University, Guiyang 550025, China College of Life Science, Guizhou University Guiyang China

**Keywords:** 16SrRNA, COI, *
Leptobrachella
*, Mayanghe National Nature Reserve, molecular phylogenetic analysis, morphology, taxonomy

## Abstract

Based on morphological comparisons and molecular phylogenetic analyses, a new species of the genus *Leptobrachella* from Mayanghe National Nature Reserve, Guizhou Province, China, is described, *Leptobrachella mayanghe***sp. nov**. Phylogenetic analyses of mitochondrial 16S rRNA and COI gene sequences supported the new species as an independent lineage. The uncorrected *p*-distances of the 16S rRNA and COI genes between the new species and its closest congeners (*L. wulingensis*, *L. yongshunensis* and *L. xinshaoensis*) are 1.8–3.4% and 8.5–9.3%, respectively. The new species is distinguished from its congeners by a combination of the following characters: (1) SVL 27.4–31.8 mm in males; (2) toes rudimentarily webbed, without lateral fringes; (3) tympanum distinct, not surrounded by prominent tubercles; (4) dorsal surface shagreened, with dense small raised tubercles and irregular longitudinal ridges; (5) ventral surface creamy white, indistinct brown markings on lateral abdomen and chest; (6) distinct black spots present on flanks; (7) heels slightly overlapping when hindlimbs are flexed at right angles to the body axis; and (8) tibia-tarsal articulation reaching the middle of the eye; (9) iris uniformly bright copper-colored. The discovery of this new species enriches the diversity of *Leptobrachella* in the Wuling Mountain range and highlights the conservation value of Mayanghe National Nature Reserve.

## Introduction

The genus *Leptobrachella* Smith, 1925, comprises small Asian leaf-litter frogs widely distributed across southern China, northeastern India, Myanmar, Indochina, the Malay Peninsula, and Borneo (Frost 2026). These species predominantly inhabit leaf litter near forest streams and exhibit high ecological and morphological diversity (Chen et al. 2018). Due to historical taxonomic controversies, members of this genus were previously assigned to multiple genera (e.g., *Paramegophrys*, *Leptolalax*; Dubois 1980, 1983), but recent integrated phylogenetic studies have unified these names under *Leptobrachella* (Chen et al. 2018).

In recent years, numerous new *Leptobrachella* species have been described in southern China, particularly in biodiversity hotspots such as the Wuling Mountains, Nanling Mountains, and Yunnan–Guizhou Plateau (Huang et al. 2025a; Liu et al. 2025; Luo et al. 2025). Guizhou Province, located in the Yunnan–Guizhou Plateau, harbors high amphibian diversity, with 11 recorded *Leptobrachella* species prior to this study. Mayanghe National Nature Reserve, situated in the northern section of the Wuling Mountains in Guizhou, features complex topography, well-developed river networks, and subtropical evergreen broad-leaved forests, providing suitable habitats for *Leptobrachella* species.

During field surveys in March 2025, we collected a series of *Leptobrachella* specimens from Mayanghe National Nature Reserve. Comprehensive morphological comparisons and molecular phylogenetic analyses revealed that this population represents an undescribed species, which we formally describe herein as *Leptobrachella mayanghe* sp. nov.

### Materials and methods

**Figure 1.**
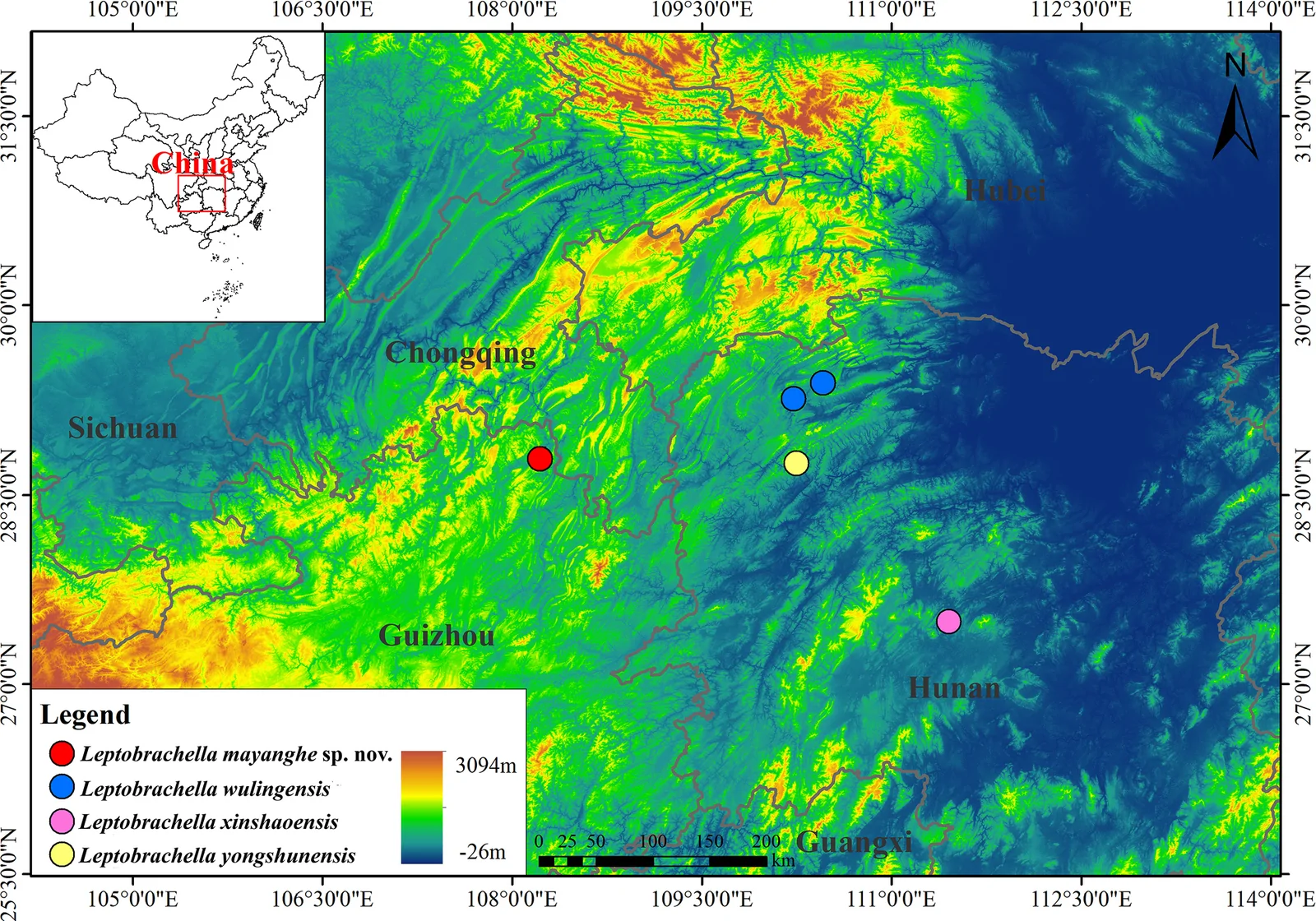
Map showing the type locality of *Leptobrachella mayanghe* sp. nov. and type localities of its three closely-related congeners in Guizhou and Hunan, China. The base maps are from the Standard Map Service website (http://bzdt.ch.mnr.gov.cn/index.html; Map Approval No. GS(2020)4619).

Seven male specimens of the new species were collected from Mayanghe National Nature Reserve (28.784573°N, 108.219349°E, ca 550 m a.s.l.), Guizhou Province, China (Table 1; Fig. 1) on 22 March 2025 by JL. All animal handling and collection procedures complied with the Wildlife Protection Law of the People’s Republic of China. All research protocols were formally approved by the Animal Care and Use Committee of Guizhou University (approval No. EAE-GZU-2024-T1228). All specimens were fixed in 10% buffered formalin for 10 h and then transferred to 75% ethanol. Tissue samples were preserved separately in 95% ethanol prior to fixation. Specimens collected in this study were deposited in the Moutai Institute (**MT**), Renhuai, Guizhou, China.

**Table 1.** Information for samples used in molecular phylogenetic analyses in this study.

**No**	**Species**	**Voucher No**.	**Locality**	**GenBank No**.	
				**16S rRNA**	**COI**
1	*L. mayanghe* sp. nov.	MT YH20250322001	Mayanghe National Nature Reserve, Guizhou, China	PZ967700	PZ979808
2	*L. mayanghe* sp. nov.	MT YH20250322002	Mayanghe National Nature Reserve, Guizhou, China	PZ967701	PZ979809
3	*L. mayanghe* sp. nov.	MT YH20250322003	Mayanghe National Nature Reserve, Guizhou, China	PZ967702	PZ979810
4	*L. mayanghe* sp. nov.	MT YH20250322004	Mayanghe National Nature Reserve, Guizhou, China	PZ967703	PZ979811
5	*L. mayanghe* sp. nov.	MT YH20250322005	Mayanghe National Nature Reserve, Guizhou, China	PZ967704	PZ979812
6	*L. mayanghe* sp. nov.	MT YH20250322006	Mayanghe National Nature Reserve, Guizhou, China	PZ967705	PZ979813
7	*L. mayanghe* sp. nov.	MT YH20250322007	Mayanghe National Nature Reserve, Guizhou, China	PZ967706	PZ979814
8	*L. xinshaoensis*	CIB XS20240319001	Xinshao County, Hunan, China	PX856296	PX852802
9	*L. xinshaoensis*	CIB XS20240319002	Xinshao County, Hunan, China	PX856297	PX852803
10	*L. xinshaoensis*	CIB XS20240319003	Xinshao County, Hunan, China	PX856298	PX852804
11	*L. wulingensis*	JWS2024422	Zhangjiajie Forest National Park, Hunan Province, China	PQ222386	PQ222459
12	*L. wulingensis*	JWS2024424	Zhangjiajie Forest National Park, Hunan Province, China	PQ222387	PQ222460
13	*L. wulingensis*	JWS2024426	Zhangjiajie Forest National Park, Hunan Province, China	PQ222388	PQ222461
14	*L. wulingensis*	CSUFT194	Tianquanshan Forest Park, Zhangjiajie, Hunan Province, China	MT530316	/
15	*L. yongshunensis*	HJ2024001	Hunan Xiaoxi National Nature Reserve, Hunan Province, China	PQ227124	PQ227123
16	*L. yongshunensis*	HJ2024004	Hunan Xiaoxi National Nature Reserve, Hunan Province, China	PQ227127	PQ227118
17	*L. yongshunensis*	HJ2024005	Hunan Xiaoxi National Nature Reserve, Hunan Province, China	PQ227128	PQ227119
18	*L. dorsospina*	SYS a004974	Yushe Forest Park, Shuicheng, Guizhou, China	MW046197	/
19	*L. purpurus*	SYS a006531	Jinzhuzhai Village, Yunnan, China	MG520355	/
20	*L. alpina*	KIZ 046816	Huangcaoling, Yunnan, China	MH055866	/
21	*L. suiyangensis*	GZNU 20180606002	Suiyang County, Guizhou, China	MK829648	/
22	*L. xishuiensis*	GZNU20240726007	Xianyuan Town, Xishui County, Guizhou	PQ604660	/
23	*L. jinshaensis*	CIBJS20200516005	Lengshuihe Nature Reserve, Jinsha County, Guizhou, China	OQ024816	OQ024417
24	*L. purpuraventra*	CIB119072	Mount Baima, Qixingguan, Bijie, Guizhou, China	OQ024811	OQ024412
25	*L. jinyunensis*	CIB119058	Mount Jinyun, Beibei District, Chongqing, China	OQ024797	OQ024396
26	*L. bijie*	CIB119068	Mount Baima, Qixingguan, Bijie, Guizhou, China	OQ024807	OQ024406
27	*L. chishuiensis*	CIBCS20190518047	Chishui National Nature Reserve, Chishui, Guizhou, China	MT117055	OQ024422
28	*L. niveimontis*	KIZ 015734	Daxueshan Nature Reserve, Yunnan, China	MT302618	/
29	*L. dong*	CIBZNY2022002	Suining County, Hunan Province, China	OP764539	PP061400
30	*L. dushanensis*	CIBDS20230310001	Dushan County, GuizhouProvince, China	PP061390	PP061404
31	*L. bourreti*	VNMN 010073	Bat Xat District, Lao Cai, Vietnam	PP061393	PP061407
32	*L. yunyangensis*	GZNU 20210629001	Lianhua Village, Yunyang County, Chongqing, China	OL800366	/
33	*L. yeae*	KIZ025778	Mount Emei, Sichuan, China	KX811928	KX812166
34	*L. graminicola*	TOLO2022.518	Mount Pu Ta Leng, Lao Cai, Vietnam	PP061392	PP061406
35	*L. eos*	NCSM 80551	Boun Tay, Phongsaly, Laos	MH055887	/
36	*L. oshanensis*	CIB EMS20190422SSG1-3	Shengshuige, Mount Emei, Sichuan, China	MT957032	/
37	*L. korifi*	KUHE 19134	Doi Inthanon, Chiang Mai, Thailand	LC741033	/
38	*L. sinorensis*	KUHE 19809	Mae Hong Son, Thailand	LC741034	/
39	*L. murphyi*	KIZ 034039	Doi Inthanon, Chiang Mai, Thailand	MZ710519	/
40	*L. tengchongensis*	SYS a003766	Gaoligong Shan, Tengchong County, Yunnan, China	MH055897	/
41	*L. mangshanensis*	MSZTC 201703	Mangshan, Hunan, China	MG132198	/
42	*L. liui*	ZYCA907	Wuyi Shan, Fujian, China	MH055908	/
43	*L. verrucosa*	GEP a062	Lianshan Bijiashan Nature Reserve, Lianshan County, Guangdong, China	OP279592	/
44	*L. dayaoshanensis*	NNU202103018	Dayaoshan, Jinxiu County, Guangxi, China	PQ476281	/
45	*L. yunkaiensis*	SYS a004663	Dawuling Forest Station, Guangdong, China	MH605584	/
46	*L. kungfu*	GEP a419	Laoxiangshan, Guangdong, China	PX431625	/
47	*L. shimentaina*	SYS a007684	Shimentai Nature Reserve, Yingde City, Guangdong, China	ON017330	/
48	*L. flaviglandulosa*	KIZ 016065	Xiaoqiaogou Nature Reserve, Yunnan, China	MT302625	/
49	*L. phiaoacensis*	IEBR A.5195	Phia Oac-Phia Den NP, Cao Bang, Vietnam	OR405871	/
50	*L. laui*	SYS a002450	Shenzhen, Guangdong, China	MH055904	/
51	*L. bashaensis*	GIB196404	Basha Nature Reserve, Guizhou, China	MW136295	/
52	*L. maoershanensis*	KIZ 07614	Mao’er Shan, Guangxi, China	MH055927	/
53	*L. khasiorum*	SDBDU 2009.329	Khasi Hills, Meghalaya, India	KY022303	/
54	*Leptolalax tamdil*	MZMU2225	NA	MW665131	/
55	*L. yingjiangensis*	SYS a006535	Jinzhuzhai Village, Yunnan, China	MG520357	/
56	*L. puhoatensis*	AMS R184852	Pu Hoat Nature Reserve, Nghe An, Vietnam	KY849588	/
57	*L. petrops*	ROM 13483	Ba Vi National Park, Ha Tay, Vietnam	MH055901	/
58	*L. namdongensis*	VNUF A.2017.37	Thanh Hoa, Vietnam	MK965389	/
59	*L. ventripunctata*	KIZ 020421	Jinuo Shan, Yunnan, China	MH055825	/
60	*L. guinanensis*	NNU 00557	Shangsi County, Guangxi, China	OP548561	/
61	*L. feii*	KIZ 048894	Xiaoqiaogou Nature Reserve, Yunnan, China	MT302634	/
62	*L. aspera*	SYS a007744	Huanglianshan Nature Reserve, Lyuchun, Yunnan, China	MW046200	/
63	*L. pelodytoides*	TZ819	NA	AF285192	/
64	*L. aerea*	NCSM 76038	Vilabuly, Savannakhet, Laos	MH055809	/
65	*L. minima*	K3124	Mae Lao Mae Sae, Doi Chiang Dao, Thailand	JN848369	/
66	*L. nyx*	AMNH A163810	Mount Tay Conn Linh, Ha Giang, Vietnam	DQ283381	/
67	*L. shangsiensis*	NHMG 1401032	Guangxi, China	MK095460	/
68	*L. phiadenensis*	IEBR A.5206	Phia Oac-Phia Den NP, Cao Bang, Vietnam	OR405873	/
69	*L. pluvialis*	MNHN 1999.5675	Sapa, Vietnam	JN848391	/
70	*L. nahangensis*	ROM 7035	Na Hang Nature Reserve, Tuyen Quang, Vietnam	MH055853	/
71	*L. shiwandashanensis*	NNU 202103213	Golden Camellia National Nature Reserve, Guangxi, China	MZ326692	/
72	*L. darongshanensis*	NNU001152	Darongshan Nature Reserve, Guangxi, China	PX480157	
73	*L. wuhuangmontis*	SYS a003485	Mt. Wuhuang, Pubei County, Guangxi, China	MH605577	/
74	*L. damingshanensis*	NNU 202103281	Damingshan National Nature Reserve, Wuming County, Guangxi, China	MZ145229	/
75	*L. wumingensis*	NNU 01058	Wuming County, Guangxi, China	OR194551	/
76	*L. yachangensis*	NHMG 250401	Yachang Orchid National Nature Reserve, Leye, Guangxi, China	PX307240	/
77	*L. batxatensis*	IEBR A.5223	Vietnam, Lao Cai Province, Bat Xat District	PP826339	/
78	*L. sungi*	ROM 20236	Tam Dao, Vinh Phuc, Vietnam	MH055858	/
79	*L. zhangyapingi*	KIZ 07258	Chiang Mai, Thailand	MH055864	/
80	*L. firthi*	AMS R 176506	Ngoc Linh Nature Reserve, Kon Tum, Vietnam	JQ739207	/
81	*L. isos*	AMS R 176469	Gia Lai, Vietnam	KT824767	/
82	*L. neangi*	CBC 1609	Phnom Samkos Wildlife Sanctuary, Veal Veng District, Pursat, Cambodia	MT644613	/
83	*L. duyenae*	IEBR A.5229	Vietnam, Khanh Hoa Province, Dien Khanh District	PP826345	/
84	*L. heteropus*	KUHE 15487	Peninsula, Malaysia	AB530453	/
85	*L. sola*	KU RMB20973	Malaysia	MH055973	/
86	*L. lateralis*	IASST AR87	India	KU674834	/
87	*Megophrys glandulosa*	KIZ048439	Yunnan Province, China	KX811762	KX812165
88	*Leptobrachium huashen*	KIZ049025	Yunnan Province, China	KX811931	KX812075

DNA was extracted from muscle tissue using a DNA extraction kit from Tiangen Biotech Co., Ltd. (Beijing). Two fragments of the mitochondrial 16S rRNA (16S) and cytochrome oxidase subunit I (COI) genes were amplified. For 16S, the primers P7 (5’-CGCCTGTTTACCAAAAACAT-3’) and P8 (5’-CCGGTCTGAACTCAGATCACGT-3’) were used (Simon et al. 1994), and for COI, Chmf4 (5’-TYTCWACWAAYCAYAAAGAYATCGG-3’) and Chmr4 (5’-ACYTCRGGRTGRCCRAARAATCA-3’) were used (Che et al. 2012). Gene fragments were amplified under the following conditions: an initial denaturing step at 95 °C for 4 min; 36 cycles of denaturing at 95 °C for 30 s, annealing at 52 °C (for 16S) or 47 °C (for COI) for 40 s, and extension at 72 °C for 70 s. Sequencing was conducted using an ABI3730 automated DNA sequencer by Shanghai DNA BioTechnologies Co., Ltd. (Shanghai, China). New sequences are deposited in GenBank (see Table 1).

For phylogenetic analyses, we downloaded corresponding sequence data of related species from GenBank (Table 1). Sequences were assembled and aligned using the ClustalW module in BioEdit v. 7.0.9.0 (Hall 1999) with default settings. Phylogenetic analyses were conducted using maximum likelihood (ML) and Bayesian inference (BI) methods, implemented in PhyML 3.0 (Guindon et al. 2010) and MrBayes v. 3.2 (Ronquist et al. 2012), respectively. The best-fit substitution model (GTR + G + I) was selected based on the Bayesian information criterion (BIC) implemented in PartitionFinder 2 (Lanfear et al. 2012). Nodal support for ML was assessed with 1000 bootstrap replicates; for BI, four Markov chains were run for 50 million generations, with the first 25% of trees discarded as burn-in. Uncorrected p-distances were calculated in MEGA v. 6.06 (Tamura et al. 2013) with 1000 bootstrap replicates.

Morphological measurements were made with dial calipers to the nearest 0.1 mm by JL, following Fei et al. (2009) and Huang et al. (2025a). Eighteen morphometric characters were measured: **ED** (eye diameter, distance from the anterior corner to the posterior corner of the eye), **FL** (foot length, distance from the tarsus to the tip of the fourth toe), **HDL** (head length, distance from the tip of the snout to the articulation of the jaw), **HDW** (maximum head width, greatest width between the left and right articulations of the jaw), **HLL** (hindlimb length, distance from the tip of the fourth toe to the vent), **IND** (internasal distance, minimum distance between the inner margins of the external nares), **IOD** (interorbital distance, minimum distance between the inner edges of the upper eyelids), **LAL** (length of the lower arm and hand, distance from the elbow to the distal end of finger IV), **LW** (lower arm width, maximum width of the lower arm), **ML** (manus length, distance from the tip of the third digit to the proximal edge of the inner palmar tubercle), **TFL** (length of the foot and tarsus, distance from the tibiotarsal articulation to the distal end of toe IV), **THL** (thigh length, distance from the vent to the knee), **TL** (tibia length, distance from the knee to the tarsus), TW (maximum tibia width), **TYD** (maximum tympanum diameter), **SL** (snout length, distance from the tip of the snout to the anterior corner of the eye), **SVL** (snout-vent length, distance from the tip of the snout to the posterior edge of the vent), and **UEW** (upper eyelid width, greatest width of the upper eyelid margins measured perpendicular to the anterior-posterior axis) (Jiang et al. 2026). Sex was determined by examination of primary sexual characteristics after dissection. No female specimens were obtained during this field survey. Comparative morphological data were obtained from the literature (Table 2).

**Table 2.** References for morphological characters for congeners of the genus *L.*.

**ID**	**Species**	**Reference**
1	*L. aerea* (Rowley, Stuart, Richards, Phimmachak & Sivongxay, 2010)	Rowley et al. 2010a
2	*L. albomarginata* Wu, Yu, Kilunda, Murphy & Che, 2025	Wu et al. 2025
3	*L. alpina* (Fei, Ye & Li, 1990)	Fei et al. 1990
4	*L. applebyi* (Rowley & Cao, 2009)	Rowley and Cao 2009
5	*L. arayai* (Matsui, 1997)	Matsui 1997
6	*L. ardens* (Rowley, Tran, Le, Dau, Peloso, Nguyen, Hoang, Nguyen & Ziegler, 2016)	Rowley et al. 2016
7	*L. aspera* Wang, Lyu, Qi & Wang, 2020	Wang et al. 2020
8	*L. aurantirosea* Ninh, Nguyen, Le, Nguyen, Quoc, Orlov, Bezman-Moseyko, Le, Nguyen & Ziegler, 2024	Ninh et al. 2024
9	*L. baluensis* Smith, 1931	Smith 1931
10	*L. bashaensis* Lyu, Dai, Wei, He, Yuan, Shi, Zhou, Ran, Kuang, Guo, Wei & Yuan, 2020	Lyu et al. 2020
11	*L. batxatensis* Hoang, Pham, Phan, Do, Wang, Jiang & Nguyen, 2025	Hoang et al. 2025
12	*L. bidoupensis* (Rowley, Le, Tran & Hoang, 2011)	Rowley et al. 2011
13	*L. bijie* Wang, Li, Li, Chen & Wang, 2019	Wang et al. 2019
14	*L. bondangensis* Eto, Matsui, Hamidy, Munir & Iskar, 2018	Eto et al. 2018
15	*L. botsfordi* (Rowley, Dau & Nguyen, 2013)	Rowley et al. 2013
16	*L. bourreti* (Dubois, 1983)	Dubois 1983
17	*L. brevicrus* Dring, 1983	Dring 1983; Eto et al. 2015
18	*L. cathaya* (Huang, Zeng, Wang, Lyu, Huang, Liu, Wang & Mo, 2026	Huang et al. 2026
19	*L. chishuiensis* Li, Liu, Wei & Wang, 2020	Li et al. 2020
20	*L. crocea* (Rowley, Hoang, Le, Dau & Cao, 2010)	Rowley et al. 2010b
21	*L. damingshanensis* Chen, Yu, Cheng, Meng, Wei, Zhou, Lu, 2021	Chen et al. 2021c
22	*L. darongshanensis* Chen, Bei & Li, 2025	Chen et al. 2025
23	*L. deocaensis* Do, Nguyen, Hoang, Ziegler & Pham, 2026	Do et al. 2026
24	*L. dayaoshanensis* Chen, Yu, Meng & Qin, 2024	Chen et al. 2024
25	*L. dong* Liu, Shi, Li, Zhang, Xiang, Wei & Wang, 2023	Liu et al. 2023
26	*L. dorsospina* Wang, Lyu, Qi & Wang, 2020	Wang et al. 2020
27	*L. dringi* (Dubois, 1987)	Dubois 1987
28	*L. dushanensis* Li, Li, Cheng, Liu, Wei, Wang, 2024	Li et al. 2024
29	*L. duyenae* Hoang, Pham, Phan, Do, Wang, Jiang & Nguyen, 2025	Hoang et al. 2025
30	*L. eos* (Ohler, Wollenberg, Grosjean, Hendrix, Vences, Ziegler & Dubois, 2011)	Ohler et al. 2011
31	*L. feii* Chen, Yuan & Che, 2020	Chen et al. 2020
32	*L. firthi* (Rowley, Hoang, Dau, Le & Cao, 2012)	Rowley et al. 2012
33	*L. flaviglandulosa* Chen, Wang & Che, 2020	Chen et al. 2020
34	*L. fritinniens* (Dehling & Matsui, 2013)	Dehling and Matsui 2013
35	*L. fuliginosa* (Matsui, 2006)	Matsui 2006
36	*L. fusca* Eto, Matsui, Hamidy, Munir & Isk & ar, 2018	Eto et al. 2018
37	*L. gracilis* (Günther, 1872)	Dehling 2012a
38	*L. graminicola* Nguyen, Tapley, Nguyen, Luong & Rowley, 2021	Nguyen et al. 2021
39	*L. guinanensis* Chen, Li, Peng, Liu & Huang, 2024	Chen et al. 2024
40	*L. hamidi* (Matsui, 1997)	Matsui 1997
41	*L. heteropus* (Boulenger, 1900)	Boulenger 1900
42	*L. huynhi* Hoang, Luong, Nguyen, Nguyen, Ninh, Le, Ziegler & Pham, 2024	Hoang et al. 2024
43	*L. isos* (Rowley, Stuart, Neang, Hoang, Dau, Nguyen & Emmett, 2015)	Rowley et al. 2015
44	*L. itiokai* Eto, Matsui & Nishikawa, 2016	Eto et al. 2016
45	*L. jinshaensis* Cheng, Shi, Li, Liu, Li & Wang, 2021	Cheng et al. 2021
46	*L. jinyunensis* Shi, Shen, Wang, Jiang & Wang, 2023	Shi et al. 2023
47	*L. juliandringi* Eto, Matsui & Nishikawa, 2015	Eto et al. 2015
48	*L. kajangensis* (Grismer, Grismer & Youmans, 2004)	Grismer et al. 2004
49	*L. kalonensis* (Rowley, Tran, Le, Dau, Peloso, Nguyen, Hoang, Nguyen & Ziegler, 2016)	Rowley et al. 2016
50	*L. kecil* (Matsui, Belabut, Ahmad & Yong, 2009)	Matsui et al. 2009
51	*L. khasiorum* (Das, Tron, Rangad & Hooroo, 2010)	Das et al. 2010
52	*L. korifi* Matsui, Panha & Eto, 2023	Matsui et al. 2023
53	*L. kungfu* Zhang, Lin, Li, Su, Chen, Zhang, Zeng & Wang, 2025	Zhang et al. 2025
54	*L. lateralis* (Anderson, 1871)	Anderson 1871; Humtsoe et al. 2008
55	*L. laui* (Sung, Yang & Wang, 2014)	Sung et al. 2014
56	*L. liui* (Fei & Ye, 1990)	Fei et al. 1990; Sung et al. 2014
57	*L. macrops* (Duong, Do, Ngo, Nguyen & Poyarkov, 2018)	Duong et al. 2018
58	*L. maculosa* (Rowley, Tran, Le, Dau, Peloso, Nguyen, Hoang, Nguyen & Ziegler, 2016)	Rowley et al. 2016
59	*L. mangshanensis* (Hou, Zhang, Hu, Li, Shi, Chen, Mo & Wang, 2018)	Hou et al. 2018
60	*L. maoershanensis* (Yuan, Sun, Chen, Rowley & Che, 2017)	Yuan et al. 2017
61	*L. marmorata* (Matsui, Zainudin & Nishikawa, 2014)	Matsui et al. 2014b
62	*L. maura* (Inger, Lakim, Biun & Yambun, 1997)	Inger et al. 1997
63	*L. melanoleuca* (Matsui, 2006)	Matsui 2006
64	*L. melica* (Rowley, Stuart, Neang & Emmett, 2010)	Rowley et al. 2010a
65	*L. minima* (Taylor, 1962)	Ohler et al. 2011; Taylor 1962
66	*L. mjobergi* Smith, 1925	Smith 1925
67	*L. murphyi* Chen, Suwannapoom, Wu, Poyarkov, Xu, Pawangkhanant & Che, 2021	Chen et al. 2021a
68	*L. nahangensis* (Lathrop, Murphy, Orlov & Ho, 1998)	Lathrop et al. 1998
69	*L. namdongensis* Hoang, Nguyen, Luu, Nguyen & Jiang, 2019	Hoang et al. 2019
70	*L. natunae* (Günther, 1895)	Günther 1895
71	*L. neangi* Stuart & Rowley, 2020	Stuart and Rowley 2020
72	*L. niveimontis* Chen, Poyarkov, Yuan & Che, 2020	Chen et al. 2020
73	*L. nokrekensis* (Mathew & Sen, 2010)	Mathew and Sen 2010
74	*L. nyx* (Ohler, Wollenberg, Grosjean, Hendrix, Vences, Ziegler & Dubois, 2011)	Ohler et al. 2011
75	*L. oshanensis* (Liu, 1950)	Liu 1950; Shi et al. 2023
76	*L. pallida* (Rowley, Tran, Le, Dau, Peloso, Nguyen, Hoang & Nguyen, 2016)	Rowley et al. 2016
77	*L. palmata* Inger & Stuebing, 1992	Inger and Stuebing 1992
78	*L. parva* Dring, 1983	Dring 1983
79	*L. pelodytoides* (Boulenger, 1893)	Boulenger 1893; Ohler et al. 2011
80	*L. petrops* (Rowley, Dau, Hoang, Le, Cutajar & Nguyen, 2017)	Rowley et al. 2017b
81	*L. phiadenensis* Luong, Hoang, Pham, Ziegler & Nguyen, 2023	Luong et al. 2023
82	*L. phiaoacensis* Luong, Hoang, Pham, Ziegler & Nguyen, 2023	Luong et al. 2023
83	*L. picta* (Malkmus, 1992)	Malkmus 1992
84	*L. pingbianensis* (Rao, Hui, Zhu & Ma, 2020)	Zhu and Rao 2020
85	*L. platycephala* (Dehling, 2012)	Dehling 2012b
86	*L. pluvialis* (Ohler, Marquis, Swan & Grosjean, 2000)	Ohler et al. 2000
87	*L. puhoatensis* (Rowley, Dau & Cao, 2017)	Rowley et al. 2017a
88	*L. purpuraventra* Wang, Li, Li, Chen & Wang, 2019	Wang et al. 2019
89	*L. purpurus* (Yang, Zeng & Wang, 2018)	Yang et al. 2018
90	*L. pyrrhops* (Poyarkov, Rowley, Gogoleva, Vassilieva, Galoyan & Orlov, 2015)	Poyarkov et al. 2015
91	*L. rowleyae* (Nguyen, Poyarkov, Le, Vo, Ninh, Duong, Murphy & Sang, 2018)	Nguyen et al. 2018
92	*L. rubromammata* Poyarkov, Nguyen, Duong, Le & Orlov, 2025	Poyarkov Jr et al. 2025
93	*L. sabahmontana* (Matsui, Nishikawa & Yambun, 2014)	Matsui et al. 2014a
94	*L. sarawakensis* Matsui, Nishikawa & Eto, 2025	Matsui et al. 2025
95	*L. serasanae* Dring, 1983	Dring 1983
96	*L. shangsiensis* Chen, Liao, Zhou & Mo, 2019	Chen et al. 2019
97	*L. shimentaina* Wang, Lyu & Wang, 2022	Wang et al. 2022
98	*L. shiwandashanensis* Chen, Peng, Pan, Liao, Liu & Huang, 2021	Chen et al. 2021b
99	*L. sinorensis* Matsui, Panha & Eto, 2023	Matsui et al. 2023
100	*L. sola* (Matsui, 2006)	Matsui 2006
101	*L. suiyangensis* Luo, Xiao, Gao & Zhou, 2020	Luo et al. 2020
102	*L. sungi* (Lathrop, Murphy, Orlov & Ho, 1998)	Lathrop et al. 1998
103	*L. tadungensis* (Rowley, Tran, Le, Dau, Peloso, Nguyen, Hoang, Nguyen & Ziegler, 2016)	Rowley et al. 2016
104	*L. tamdil* (Sengupta, Sailo, Lalremsanga, Das & Das, 2010)	Sengupta et al. 2010
105	*L. tengchongensis* (Yang, Wang, Chen & Rao, 2016)	Yang et al. 2018
106	*L. tuberosa* (Inger, Orlov & Darevsky, 1999)	Inger et al. 1999
107	*L. ventripunctata* (Fei, Ye & Li, 1990)	Fei et al. 1990
108	*L. verrucosa* Wang, Zeng, Lin & Li, 2022	Lin et al. 2022
109	*L. weixinensis* Liu, Chen, Xu & Wu, 2025	Liu et al. 2025
110	*L. wuhuangmontis* Wang, Yang & Wang, 2018	Wang et al. 2018
111	*L. wulingensis* Qian, Xiao, Cao, Xiao & Yang, 2020	Qian et al. 2020
112	*L. wumingensis* Chen, Peng, Li & Yu, 2023	Chen et al. 2023
113	*L. xinshaoensis* Jiang, Liu, Huang, Zhang & Wang, 2026	Jiang et al. 2026
114	*L. xishuiensis* Luo, Deng, Zhou, 2025	Luo et al. 2025
115	*L. yachangensis* Huang, Zeng, Wang, Wang, Deng, Dong, Liao & Mo, 2025	Huang et al. 2025b
116	*L. yeae* Shi, Hou, Song, Jiang & Wang, 2021	Shi et al. 2021
117	*L. yingjiangensis* (Yang, Zeng & Wang, 2018)	Yang et al. 2018
118	*L. yongshunensis* Huang, Wu, Jiang & Zhang, 2025	Huang et al. 2025a
119	*L. yunkaiensis* Wang, Li, Lyu & Wang, 2018	Wang et al. 2018
120	*L. yunyangensis* Luo, Deng & Zhou, 2022	Luo et al. 2022
121	*L. zhangyapingi* (Jiang, Yan, Suwannapoom, Chomdej & Che, 2013)	Jiang et al. 2013

## Results

**Figure 2.**
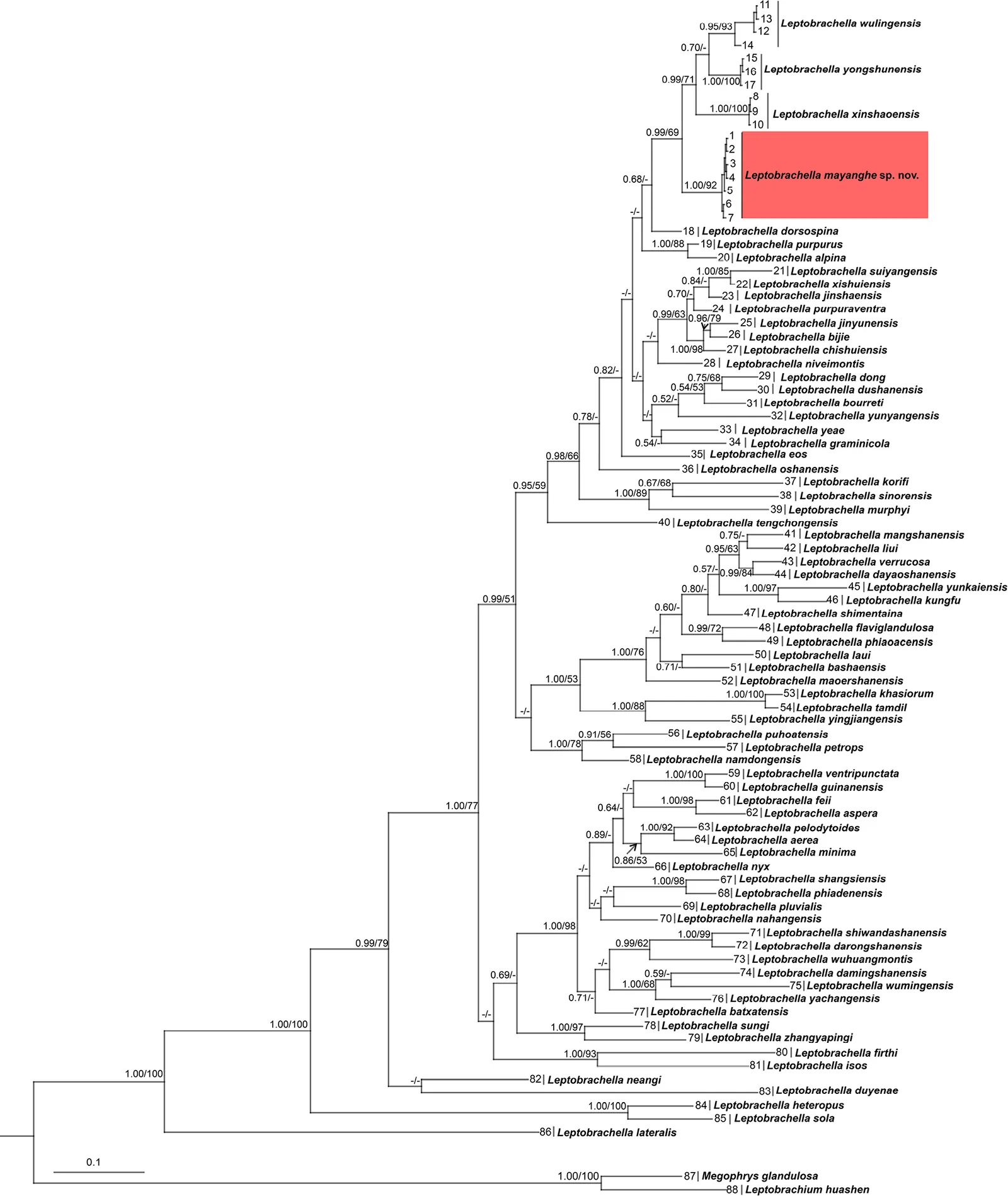
ML tree of the genus *Leptobrachella* reconstructed based on the 16S and COI gene sequences. The new species is indicated in 1-7. Bayesian posterior probabilities and ML bootstrap support values are shown next to each node, and “-” denotes BS < 50% or BPP < 0.50. Samples 1–88 refer to Table 1.

A total of 501 bp of the 16S gene and 603 bp of the COI gene were concatenated. Maximum likelihood (ML) and Bayesian inference (BI) trees showed consistent topologies, with strong support for major nodes. All samples of the new species clustered into a distinct clade (BPP = 1.00; BS = 92%), sister to a clade comprising *L. wulingensis*, *L. yongshunensis*, and *L. xinshaoensis* (BPP = 0.99; BS = 69%; Fig. 2).

Based on the 16S and COI genes, the minimum interspecific genetic distances between *L. mayanghe* sp. nov. and its congeners are as follows: 1.8% vs *L. wulingensis*, 2.4% vs *L. yongshunensis* and 3.4% vs *L. xinshaoensis* for the 16S gene; 8.5% vs *L. wulingensis*, 9.0% vs *L. yongshunensis* and 9.3% vs *L. xinshaoensis* for the COI gene. These values meet or exceed the levels of divergence observed between most valid species pairs within the genus (e.g., only 3.8% for the COI gene between *L. bijie* and *L. jinshaensis*; Suppl. materials 1, 2). Furthermore, these specimens can be distinguished from all other congeners within the genus by a diagnostic combination of morphological characters (see taxonomic account below).

Comparative morphological data of the undescribed taxon and 93 recognized *Leptobrachella* species occurring north of the Isthmus of Kra are listed in Suppl. material 3. Among the 27 congeners of the genus *Leptobrachella* occurring south of the Isthmus of Kra, by the presence of supra-axillary and ventrolateral glands, the undescribed taxon is readily distinguished from *L. arayai*, *L. dringi*, *L. fritinniens*, *L. gracilis*, *L. hamidi*, *L. heteropus*, *L. kajangensis*, *L. kecil*, *L. marmorata*, *L. melanoleuca*, *L. maura*, *L. picta*, *L. platycephala*, *L. sabahmontana*, *L. sarawakensis*, and *L. sola* (vs absent in the others). By having a moderate body size (SVL 27.35–31.75 mm in males), the new species differs from *L. applebyi*, *L. aspera*, *L. baluensis*, *L. brevicrus*, *L. bondangensis*, *L. feii*, *L. fusca*, *L. itiokai*, *L. juliandringi*, *L. melica*, *L. mjobergi*, *L. natunae*, *L. niveimontis*, *L. palmata*, *L. parva*, *L. pluvialis*, *L. rubromammata*, and *L. serasanae* (vs less than 24 mm in the males or females); and from the larger species *L. deocaensis*, *L. nahangensis*, *L. sungi*, and *L. zhangyapingi* (vs larger than 35 mm in these).

By the presence of black spots on flanks, the undescribed taxon differs from *L. aerea*, *L. botsfordi*, *L. crocea*, *L. deocaensis*, *L. eos*, *L. firthi*, *L. isos*, *L. juliandringi*, *L. pallida*, *L. petrops*, and *L. tuberosa* (vs absent).

By the rudimentary webbing on the toes, the new species differs from *L. ardens*, *L. aurantirosea*, *L. batxatensis*, *L. deocaensis*, *L. duyenae*, *L. huynhi*, *L. jinshaensis*, *L. kalonensis*, *L. maculosa*, *L. oshanensis*, *L. pallida*, *L. petrops*, *L. rowleyae*, *L. rubromammata*, *L. shiwandashanensis*, *L. tadungensis*, *L. verrucosa*, *L. wumingensis*, and *L. yachangensis* (vs absent); from *L. sungi* and *L. tamdil* (vs wide); and from *L. guinanensis* and *L. pelodytoides* (vs 1/3 toe webbing).

By the lateral fringes on toes absent, the new species differs from *L. aerea*, *L. alpina*, *L. dayaoshanensis*, *L. dong*, *L. dushanensis*, *L. eos*, *L. firthi*, *L. graminicola*, *L. guinanensis*, *L. isos*, *L. khasiorum*, *L. kungfu*, *L. laui*, *L. liui*, *L. murphyi*, *L. purpurus*, *L. shimentaina*, *L. tamdil*, *L. yingjiangensis*, *L. yunkaiensis*, and *L. zhangyapingi* (vs wide); and from *L. albomarginata*, *L. aspera*, *L. aurantirosea*, *L. bashaensis*, *L. batxatensis*, *L. bijie*, *L. botsfordi*, *L. chishuiensis*, *L. damingshanensis*, *L. dorsospina*, *L. feii*, *L. flaviglandulosa*, *L. huynhi, L. jinshaensis*, *L. jinyunensis*, *L. mangshanensis*, *L. maoershanensis*, *L. pelodytoides*, *L. petrops*, *L. puhoatensis*, *L. purpuraventra*, *L. shangsiensis*, *L. suiyangensis*, *L. tengchongensis*, *L. verrucosa*, *L. weixinensis*, *L. wuhuangmontis*, *L. wulingensis*, *L. xinshaoensis*, *L. yachangensis*, *L. yeae*, *L. yongshunensis*, and *L. yunyangensis* (vs narrow).

By the tibia-tarsal articulation reaching the middle of eye the undescribed taxon differs from *L. chishuiensis* (vs reaching the tympanum), *L. dorsospina*, *L. nokrekensis*, and *L. jinyunensis* (vs reaching the posterior corner of eye), *L. alpina*, *L. aspera*, *L. korifi*, *L. laui*, *L. neangi*, *L. purpurus*, *L. suiyangensis*, *L. verrucosa*, and *L. yunyangensis* (vs reaching just past the anterior edge of the eye), *L. ventripunctata* and *L. xishuiensis* (vs reaching between eye and tympanum), *L. bashaensis*, *L. mangshanensis*, *L. maoershanensis*, *L. pluvialis*, *L. sinorensis*, and *L. weixinensis* (vs reaching the nostril).

By heels overlapping when hind-limbs flexed at right angles to the axis of body the undescribed taxon differs from *L. guinanensis*, *L. phiadenensis*, *L. phiaoacensis*, *L. shiwandashanensis*, *L. verrucosa*, and *L. xinshaoensis* (vs heels not meeting).

The undescribed taxon differs from 19 congeneric species (*L. aerea*, *L. albomarginata*, *L. alpinus*, *L. applebyi*, *L. ardens*, *L. aspera*, *L. aurantirosea*, *L. bashaensis*, *L. batxatensis*, *L. bidoupensis*, *L. bijie*, *L. bourreti*, *L. fuliginosa*, *L. mangshanensis*, *L. pingbianensis*, *L. shangsiensis*, *L. ventripunctata*, *L. yeae*) by two diagnostic character combinations: dorsal skin texture: shagreened with small, raised, orange tubercles, and distinct dark brown blotches/longitudinal ridges (opposing states in the compared species range from completely smooth, nearly smooth with few tubercles, finely tuberculate without shagreened texture, to rough with dense conical granules/large warts; none exhibit the combination of dense raised, orange tubercles and prominent dark blotches/longitudinal ridges); ventral coloration: creamy white with dense dark brown spots on the chest and lateral margins (opposing states include uniformly creamy white without dark spots, reddish brown with white speckling, creamy white with full-venter marbling, greyish white with dark grey spots, yellowish creamy white with marble texture, and white with only faint grey/brown speckling; none exhibit the dense dark brown spots restricted to the chest and lateral margins).

The new taxon can clearly be distinguished from its phylogenetically close congeners *L. wulingensis*, *L. xinshaoensis*, and *L. yongshunensis* (Fig. 5). It differs from *L. wulingensis* by the lateral fringes on toes absent (vs lateral fringes on toes narrow); iris uniformly bright copper-colored (vs iris bicolored, golden in the upper half, and fade to dark silver in lower half); dorsal skin shagreened with small tubercles and ridge (vs shagreened with sparse large warts, sometimes with longitudinal ridges); differs from *L. xinshaoensis* by the lateral fringes on toes absent (vs lateral fringes on toes narrow); heels overlapping when hind-limbs flexed at right angles to the axis of body (vs no meeting); the tibia-tarsal articulation reaching the middle of eye (vs reaching the eye and the tympanum); differs from *L. yongshunensis* by the lateral fringes on toes absent (vs lateral fringes on toes narrow); the tibia-tarsal articulation reaching the middle of eye (vs reaching the anterior edge of the eye); iris uniformly bright copper-colored (vs iris bicolored, coppery in the upper half, and fading to dark silver in lower half); dorsal skin shagreened with small tubercles and ridge (vs shagreened with sparse large warts and dense little wart grains, sometimes with longitudinal ridges).

### Leptobrachella mayanghe sp. nov.

Taxon classificationAnimaliaAnuraMegophryidae

ADFE15DC-7B4D-53A5-B075-B5A970EA4B1D

https://zoobank.org/9BEE97A6-1CB2-42FA-B4B6-F2191843C0C5

Figs 3, 4, 5, 6

#### Type material.

**Figure 3.**
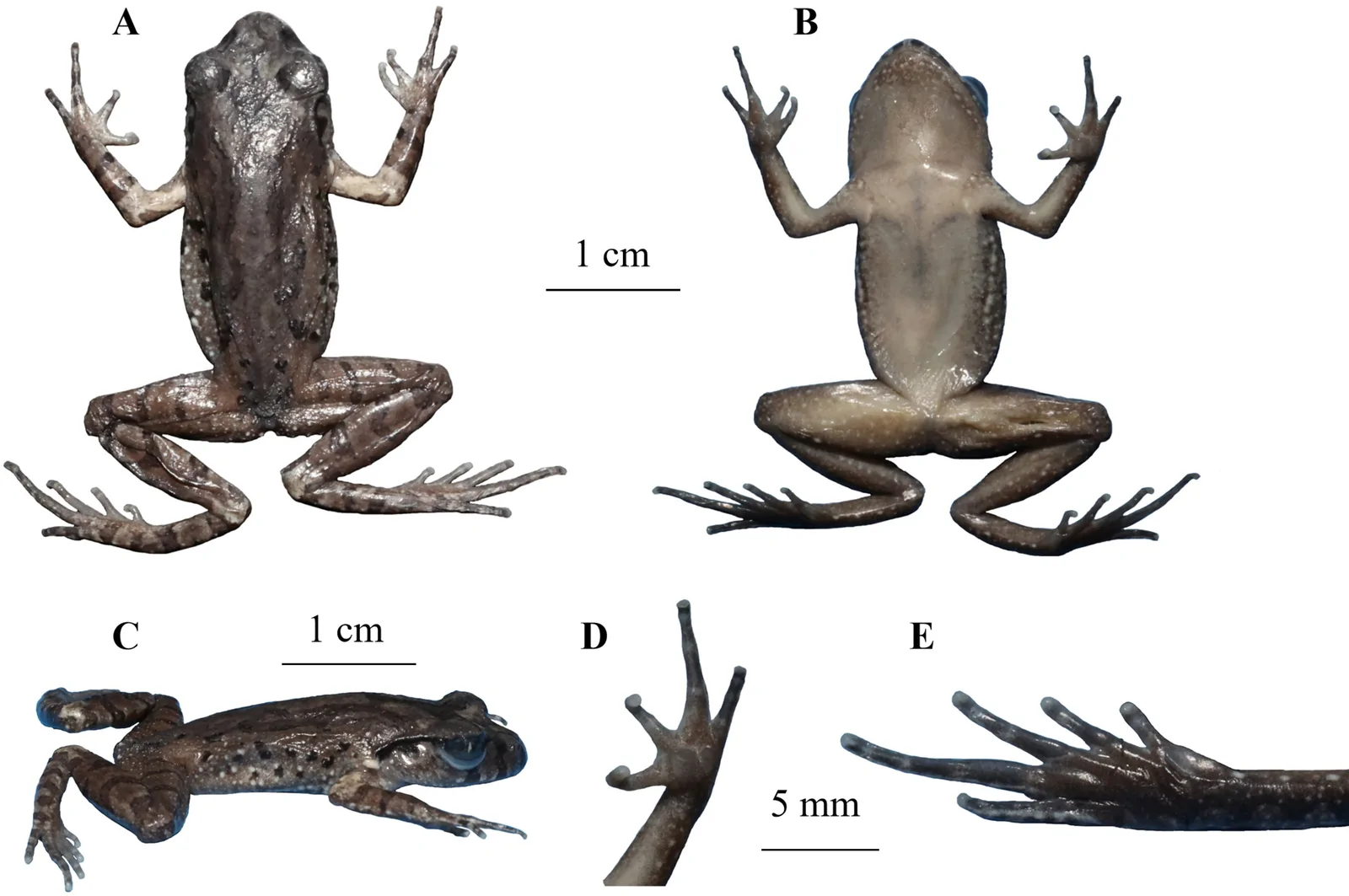
The holotype specimen MT YH20250322007 of *Leptobrachella mayanghe* sp. nov. (preserved) in **A**. Dorsal view; **B**. Ventral view; **C**. Lateral view; **D**. Ventral view of hand; **E**. Ventral view of foot. Photographs by SL.

**Figure 4.**
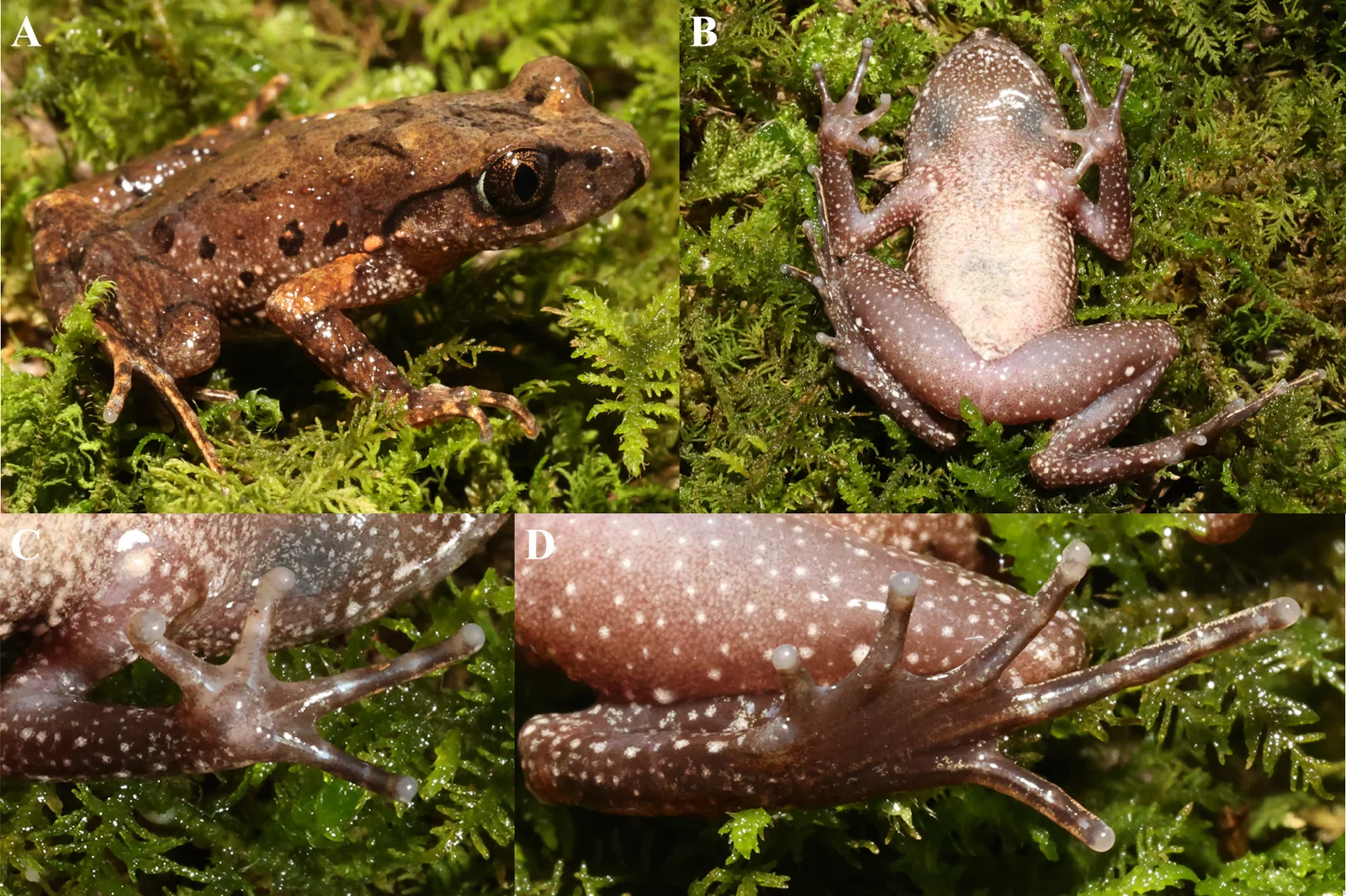
Photographs of the holotype MT YH20250322007 of *Leptobrachella mayanghe* sp. nov. in life. **A**. Dorsal view; **B**. Ventral view; **C**. Ventral view of hand; **D**. Ventral view of foot. Photographs by SL.

***Holotype***. MT YH20250322007 (Figs 3, 4), • adult male, collected from Mayanghe National Nature Reserve, Guizhou Province, China (28.784573°N, 108.219349°E, ca 550 m a.s.l.), collected by JL on 22 March 2025. ***Paratypes***. • Six adult males, MT YH20250322001–006, collected from the same locality as the holotype on 22 March 2025 by SL.

#### Diagnosis.

*Leptobrachella mayanghe* sp. nov. can be distinguished from its congeners by a combination of the following characters: (1) SVL 27.4–31.8 mm in males; (2) toes rudimentarily webbed, without lateral fringes; (3) tympanum distinct, not surrounded by prominent tubercles; (4) dorsal surface shagreened, with dense small raised tubercles and irregular longitudinal ridges; (5) ventral surface creamy white, indistinct brown markings on lateral abdomen and chest; (6) distinct black spots present on flanks; (7) heels slightly overlapping when hindlimbs are flexed at right angles to the body axis; (8) tibia-tarsal articulation reaching the middle of the eye; (9) iris uniformly bright copper-colored.

#### Description of the holotype.

Adult male, SVL 30.1 mm; head length (HDL 10.4 mm) slightly greater than head width (HDW 9.8 mm); snout rounded in dorsal and lateral views, projecting slightly beyond the lower jaw; nostril oval, closer to snout tip than to eye (SL 4.4 mm, IND 3.8 mm); loreal region oblique and slightly concave; interorbital distance (IOD 3.4 mm) narrower than internarial distance; eyes large and protuberant, pupil vertical, iris uniformly bright copper-colored; tympanum rounded, diameter (TYD 2.8 mm) beyond half the eye diameter (ED 3.9 mm), supratympanic ridge distinct from posterior corner of eye to supra-axillary gland; tongue notched behind; vomerine teeth absent.

Forelimbs slender, LAL / SVL = 0.51; relative finger length I < II < IV < III; finger tips rounded and slightly swollen; subarticular tubercles indistinct; inner metacarpal tubercle large and round, outer metacarpal tubercle small and flattened, distinctly separated; finger webbing absent, lateral fringes absent (Figs 3, 4).

Hindlimbs long, hindlimb length (HLL 45.7 mm) 1.52 times the snout-vent length (SVL 30.1 mm); thigh length (THL 12.6 mm) slightly shorter than tibia length (TL 14.6 mm); tibia width (TW 3.3 mm) 22.5% of tibia length (TL 14.58 mm); relative toe length I < II < V < III < IV; toes with rudimentary webbing and lateral fringes absent; inner metatarsal tubercle elongated, outer metatarsal tubercle absent; tibia-tarsal articulation reaching the middle of eye; heels overlapping when hind-limbs flexed at right angles to the axis of body (Figs 3, 4).

Dorsal surface shagreened, with dense small raised tubercles and irregular longitudinal ridges; flanks with scattered large glandular warts interspersed with black spots; ventral surface smooth, chest and abdomen creamy white; pectoral glands oval, femoral glands oval (closer to knee than vent); supra-axillary gland small and rounded, ventrolateral glandular line distinct.

Dorsum with sparse large warts, dense fine wart granules and ridges; upper eyelids, tympanic region and dorsal surfaces of limbs densely covered with small wart granules; flanks with large glandular warts, ventrolateral glandular line is indistinct; white conical spines present on lateral and ventral surfaces of tarsus, surface of tibiotarsal joint, inner side of shank and around cloacal region; pectoral glands and femoral glands oval; femoral glands situated on posteroventral surface of thigh, closer to knee than to vent; supra-axillary gland prominently raised, rounded, and orange in life; ventrolateral glandular line distinctly visible.

#### Coloration of holotype in life.

**Figure 5.**
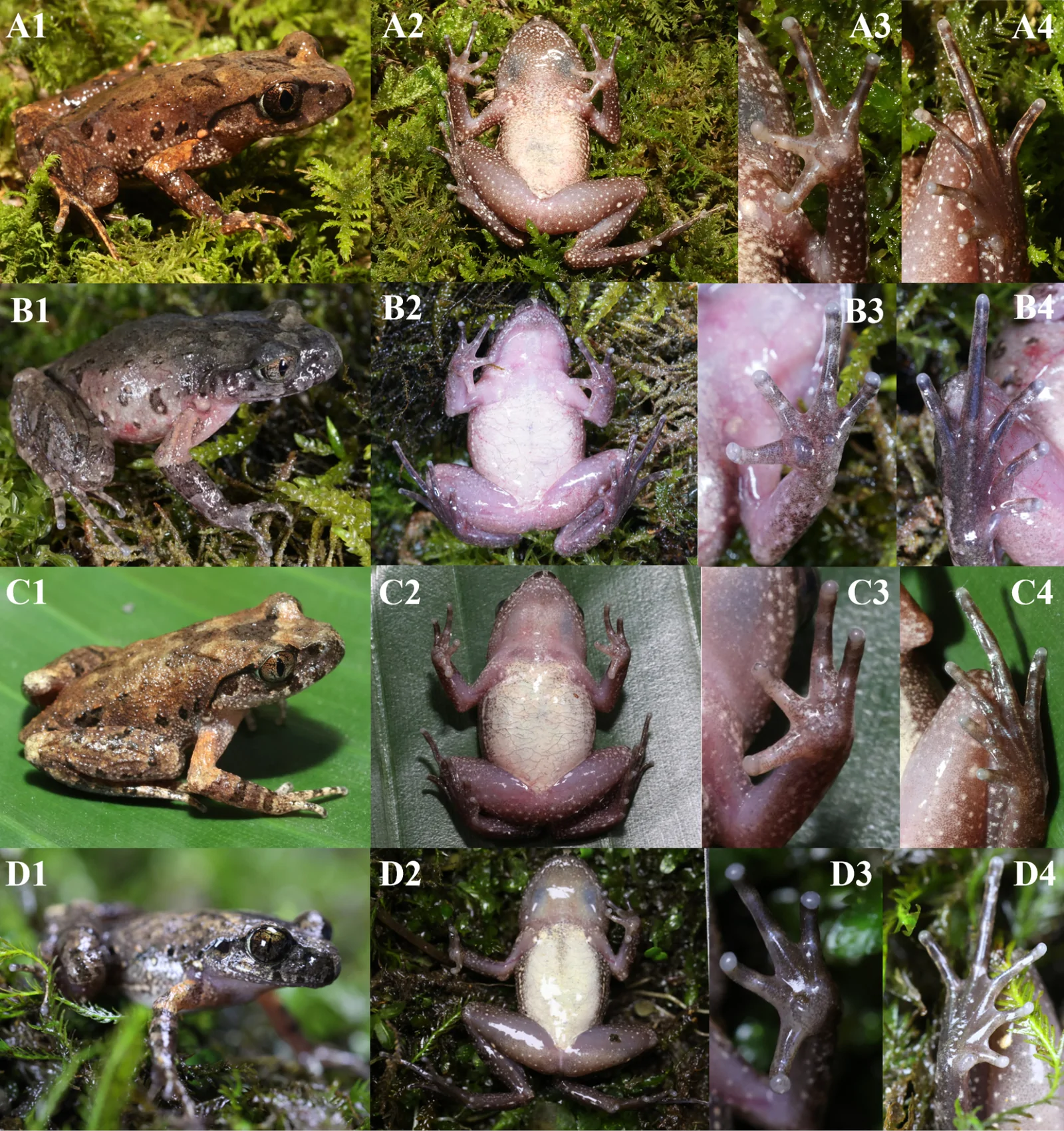
Comparative morphological photographs of closely related *Leptobrachella* species. **A**. *Leptobrachella mayanghe* sp. nov.; **B**. *Leptobrachella wulingensis*; **C**. *Leptobrachella xinshaoensis*; **D**. *Leptobrachella yongshunensis*. Photos **A, C**. by SL; B1–B4 by SS; D1–D4 by JH.

Dorsal ground color brownish, with an inverted brown triangular marking between the eyes and a W-shaped marking on the anterior mid-dorsum. Flanks with black spots interspersed with two rows of large warts. A conspicuous orange patch is present on the upper arm near the axilla; lower part of forelimbs with black transverse bars; hindlimbs with black transverse bars; fingers and toes all with transverse bars (Figs 4, 5A). A pale brown longitudinal stripe below the tympanic region; margin of tympanum darkly pigmented.

Ventral surface mainly creamy white, with indistinct brown markings on lateral abdomen and chest; flanks, ventral surfaces of limbs and toes with white translucent spots (Figs 4, 5A). Supra-axillary gland pale orange; pectoral glands and femoral glands creamy white.

#### Coloration of holotype in preservation.

In 75% ethanol, the dorsal and limb surfaces have faded to a uniform dark brown; the brown inverted triangular marking between the eyes remains distinctly visible. Irregular black spots on the flanks are clearly defined, while dorsolateral markings, longitudinal skin ridges, and dorsal spots have become more prominent against the faded background. The throat, chest, and belly are creamy white, with the pectoral, femoral, supra-axillary, and ventrolateral glands also appearing creamy white. Dark transverse bars on the limbs, fingers, and toes remain distinct, while the upper arm and tibiotarsus have faded to a greyish-white. The iris is uniformly dark grey.

#### Variation.

Measurements of the type series are summarized in Table 3. All paratypes conform to the holotype in general morphology with minor variations: SVL ranges from 27.4 mm (MT YH20250322006) to 31.8 mm (MT YH20250322004).

**Table 3.** Measurements of the seven adult male specimens of *Leptobrachella mayanghe* sp. nov. Units are given in mm. See abbreviations for the morphological characters in Materials and methods section.

**Voucher**	**MT YH20250322001**	**MT YH20250322002**	**MT YH20250322003**	**MT YH20250322004**	**MT YH20250322005**	**MT YH20250322006**	**MT YH20250322007**	**Range**	**Mean ± SD**
**Sex**	**Male**	**Male**	**Male**	**Male**	**Male**	**Male**	**Male**	**Males (*n* = 7)**	
ED	4.4	4.4	4.0	4.5	4.4	4.3	3.9	3.9–4.5	4.3 ± 0.2
FL	15.3	14.7	13.4	15.5	13.6	14.5	14.7	13.4–15.5	14.5 ± 0.8
HDL	10.3	10.6	9.9	10.6	9.7	10.0	10.4	9.7–10.6	10.2 ± 0.4
HDW	10.1	10.0	9.7	10.6	9.3	9.3	9.8	9.3–10.6	9.8 ± 0.5
HLL	46.5	43.7	42.1	46.3	42.0	41.1	45.7	41.1–46.5	43.9 ± 2.3
IND	4.5	4.3	3.5	3.8	4.1	3.7	3.8	3.5–4.5	4.0 ± 0.4
IOD	2.8	3.2	3.0	3.2	2.4	3.4	3.4	2.4–3.4	3.1 ± 0.4
LAL	14.5	14.7	13.2	15.4	14.4	14.0	15.3	13.2–15.4	14.5 ± 0.8
LW	2.4	1.9	1.7	2.0	1.8	1.9	1.9	1.7–2.4	2.0 ± 0.2
ML	8.4	8.2	7.5	8.6	7.8	8.0	8.1	7.5–8.6	8.1 ± 0.4
SL	5.1	4.4	4.3	5.6	4.3	4.4	4.4	4.3–5.6	4.6 ± 0.5
SVL	30.7	30.6	27.5	31.8	29.2	27.4	30.1	27.4–31.8	29.6 ± 1.7
TFL	20.6	20.5	18.3	20.3	19.7	19.9	20.9	18.3–20.9	20.0 ± 0.9
THL	14.3	13.5	12.9	13.2	12.9	10.6	12.6	10.6–14.3	12.9 ± 1.2
TL	14.6	13.7	13.2	14.9	14.0	13.2	14.6	13.2–14.9	14.0 ± 0.7
TW	3.4	3.5	2.8	3.2	2.9	2.3	3.3	2.3–3.5	3.1 ± 0.4
TYD	2.1	2.9	2.2	2.3	2.4	2.5	2.8	2.1–2.9	2.5 ± 0.3
UEW	3.6	3.3	2.8	3.0	3.4	3.0	2.7	2.7–3.6	3.1 ± 0.3

**Figure 6.**
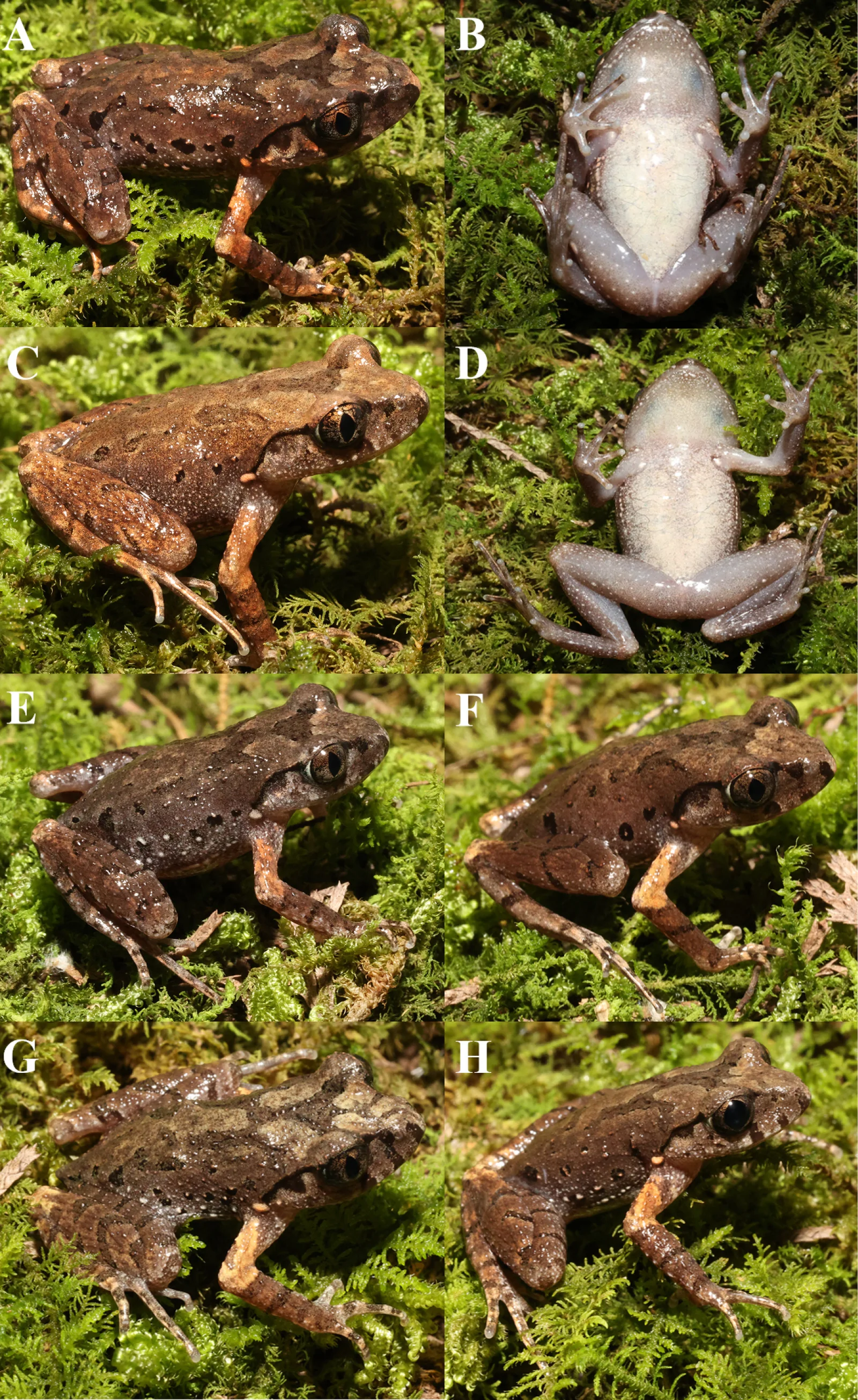
Color variation in *Leptobrachella mayanghe* sp. nov. **A**. Dorsolateral view and **B**. Ventral view of the specimen MT YH20250322001; **C**. Dorsolateral view; **D**. Ventral view of the specimen MT YH20250322002; **E**. Dorsolateral view of the specimen MT YH20250322003; **F**. Dorsolateral view of the specimen MT YH20250322006; **G**. Dorsolateral view of the specimen MT YH20250322005; **H**. Dorsolateral view of the specimen MT YH20250322004. Photos **A–H**. by SL.

***Dorsal coloration***: the dorsal ground color varies from pale brown to dark brown. Some individuals (e.g., Fig. 6E, G, H) exhibit a distinctly darker, almost blackish-brown tone, while others (e.g., Fig. 6C) show a paler, more uniform brown. The density and shape of dark dorsal spots also differ: some specimens have larger, irregular blotches, while others have smaller, scattered spots. The orange pigmentation on the upper arms near the axilla is more conspicuous in some individuals (e.g., Fig. 6A, H) and paler in others (e.g., Fig. 6C, F).

***Ventral coloration***: specimen MT YH20250322001(Fig. 6B) displays a distinctly whiter abdominal coloration with minimal brown markings. In contrast, the posterior margin of the abdomen in specimen MT YH20250322002 (Fig. 6D) is pale purple, contrasting with the creamy white base. Other paratypes show ventral surfaces ranging from creamy white with fine white granules (Fig. 6B, D) to pale brown with more prominent brown markings along the abdominal margins.

#### Secondary sexual characters.

Adult males with subgular vocal sacs, femoral adipose glands present on posterior surface of thigh. Nuptial pads and spines absent on males.

#### Distribution and habitats.

**Figure 7.**
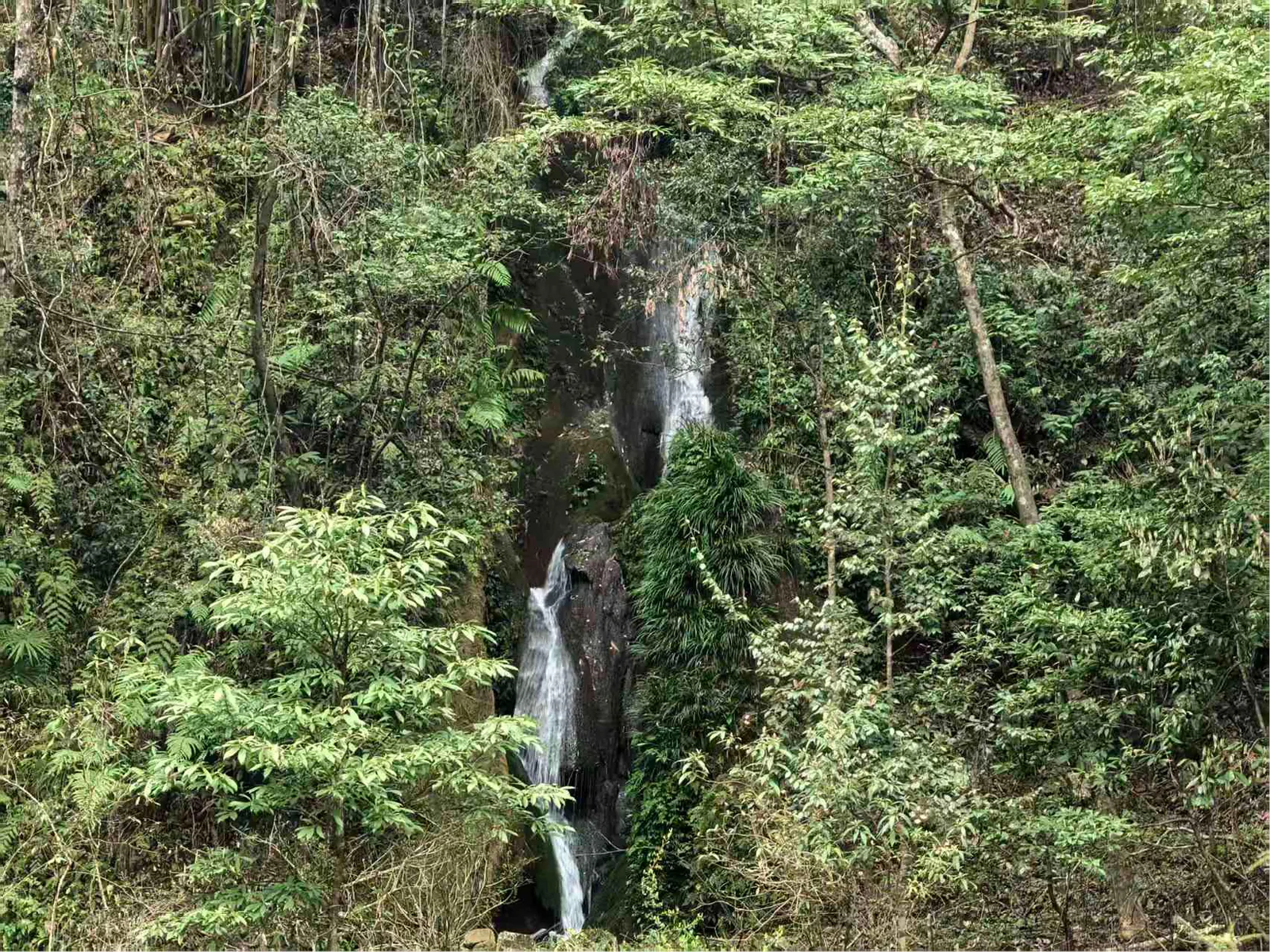
Habitats of *Leptobrachella mayanghe* sp. nov. at the type locality, Mayanghe National Nature Reserve, Yanhe County, Guizhou Province, China. Photo by JL.

*Leptobrachella mayanghe* sp. nov. is currently known only from its type locality, Mayanghe National Nature Reserve, Guizhou Province, China. Specimens were found on rocks or plant leaves near waterfalls of clear perennial mountain streams (Fig. 7). The habitat is characterized by subtropical evergreen broad-leaved forest with dense understory vegetation. No females, eggs, or tadpoles were collected. Based on our subsequent field surveys, advertisement calls of this new species could be heard from March to May. Therefore, the breeding season is inferred to be March-May. Sympatric amphibian species include *Leptobrachium boringii* (Liu, 1945) and *Quasipaa boulengeri* (Günther, 1889).

#### Etymology.

The specific epithet *mayanghe* refers to the type locality, Mayanghe National Nature Reserve, Guizhou Province, China. We suggest the English name “Mayanghe Leaf-litter Toad” and the Chinese name “Má Yáng Hé Zhǎng Tū Chán (麻阳河掌突蟾)”.

## Discussion

Integrative taxonomic evidence from mitochondrial DNA and morphological characters consistently supports *Leptobrachella mayanghe* sp. nov. as a valid and evolutionarily distinct species within the Wuling Mountains clade of the genus *Leptobrachella*, which aligns with the well-documented biogeographic pattern of *Leptobrachella* diversification in this region.

Mayanghe National Nature Reserve is located in the Wuling Mountain biodiversity hotspot and serves as the habitat for numerous endemic species. The discovery of *Leptobrachella mayanghe* sp. nov. further confirms the exceptionally high conservation value of this karst region, which is recognized as a critical refugium for amphibian diversity in southern China (Luo et al. 2016). Given its restricted distribution and dependence on stream-associated habitats, the new species is vulnerable to habitat disturbance. The species is currently known only from its type locality and relies on specific habitats, while amphibians are facing global threats such as habitat loss, climate change, and emerging diseases (Stuart et al. 2010). It is recommended that its conservation status be assessed according to the IUCN Red List criteria (IUCN 2022), and the protection of its habitat be strengthened, particularly focusing on the preservation of stream integrity and riparian vegetation, which are essential for the survival of *Leptobrachella* species. Furthermore, the discovery of *L. mayanghe* sp. nov. further demonstrates the biodiversity potential of karst regions, which may harbor more undiscovered cryptic species due to their complex geological and microhabitat conditions (Che et al. 2018). We recommend conducting more systematic biodiversity surveys and monitoring in this region to better understand the regional background values and biological resources, which is crucial for formulating effective conservation strategies for amphibians in karst ecosystems (Luo et al. 2016).

## Supplementary Material

XML Treatment for Leptobrachella mayanghe

## Additional information

### Artificial Intelligence (AI) use

The authors accept full responsibility for the content of the manuscript, including the disclosure of any use of AI.

No AI tools were used in the preparation of this manuscript.

### Author ORCIDs

J. Liu https://orcid.org/0000-0001-9579-324X

### Data availability

All of the data that support the findings of this study are available in the main text or Supplementary Information.

## Supplementary materials

Supplementary material 1 Uncorrected *p*-distances between the *Leptobrachella* species based on the 16S gene sequences in this study. Data type xls https://binary.pensoft.net/file/1813611This dataset is made available under the Open Database License (http://opendatacommons.org/licenses/odbl/1.0/). The Open Database License (ODbL) is a license agreement intended to allow users to freely share, modify, and use this Dataset while maintaining this same freedom for others, provided that the original source and author(s) are credited. Zhaohui Yang, Jing Liu, Fan Zhang, Yanlin Cheng, Qixian Zou, Yunlong Chen, Lang Mu, Junjian Zhou, Shize Li, Heng Zhao

Supplementary material 2 Uncorrected *p*-distances between the *Leptobrachella* species based on the COI gene sequences in this study Data type xls https://binary.pensoft.net/file/1813612This dataset is made available under the Open Database License (http://opendatacommons.org/licenses/odbl/1.0/). The Open Database License (ODbL) is a license agreement intended to allow users to freely share, modify, and use this Dataset while maintaining this same freedom for others, provided that the original source and author(s) are credited. Zhaohui Yang, Jing Liu, Fan Zhang, Yanlin Cheng, Qixian Zou, Yunlong Chen, Lang Mu, Junjian Zhou, Shize Li, Heng Zhao

Supplementary material 3 Selected diagnostic characters for species described herein and species in the genus *Leptobrachella* Data type xls https://binary.pensoft.net/file/1813613This dataset is made available under the Open Database License (http://opendatacommons.org/licenses/odbl/1.0/). The Open Database License (ODbL) is a license agreement intended to allow users to freely share, modify, and use this Dataset while maintaining this same freedom for others, provided that the original source and author(s) are credited. Zhaohui Yang, Jing Liu, Fan Zhang, Yanlin Cheng, Qixian Zou, Yunlong Chen, Lang Mu, Junjian Zhou, Shize Li, Heng Zhao
